# Improvement of left ventricular systolic function in morbidly obese patients after bariatric surgery: Case report

**DOI:** 10.1097/MD.0000000000024309

**Published:** 2021-02-12

**Authors:** Yanjun Liu, Pengsen Guo, Dafang Zhan, Luo Fu, Jiahui Yu, Huawu Yang

**Affiliations:** a Affiliated to Southwest Jiaotong University Obesity and Metabolism Center, Third People's Hospital of Chengdu; b Southwest Jiaotong University, Chengdu, China.

**Keywords:** bariatric surgery, case report, heart failure, left ventricular systolic dysfunction, morbid obesity

## Abstract

**Introduction:**

Morbid obesity (body mass index > 40 kg/m2) is a risk factor for the development of left ventricular systolic dysfunction (LVSD) and can complicate the management of LVSD. Bariatric surgery is increasingly recognized as a safe and effective way to achieve marked weight loss, but studies on improving LVSD populations are limited. We retrospectively analyzed the first case of the Asia-Pacific region with morbid obesity and left ventricular ejection fraction (LVEF) < 50% who underwent bariatric surgery at our medical center.

**Patient concerns:**

The patient was admitted to the hospital due to progressive weight gain for more than 10 years. The patient used to be in good health. One year before admission, the patient was hospitalized in another hospital due to shortness of breath. After the relevant examination, the patient was diagnosed with dilated cardiomyopathy.

**Diagnosis:**

The body mass index of the patient was 45.9 kg/m^2^, and the patient was diagnosed with morbid obesity. He was diagnosed with dilated cardiomyopathy and cardiac function class IV in another hospital. After completing a preoperative examination, the patient was diagnosed with hyperuricemia, hyperlipidemia, fatty liver disease and severe sleep apnea.

**Interventions:**

The patient successfully underwent laparoscopic sleeve gastrectomy plus jejunal bypass.

**Outcomes:**

Six months after the surgery, patient weight lost was 33.6 kg, and the LVEF increased from 31% to 55%. The cardiac function of the patient recovered from class IV to class I, and the patient's hyperuricemia, hyperlipidemia and sleep apnea were significantly improved.

**Conclusion:**

Bariatric surgery may be a safe and effective intervention for morbidly obese patients with LVSD. Bariatric surgery was associated with an improvement in LVEF. However, the specific mechanism still needs further study.

## 1 Introduction

Morbid obesity is an increasingly prevalent chronic metabolic disease that is associated with multiple comorbidities, such as hypertension, diabetes, dyslipidemia, and heart disease.^[1,2]^ In the general population, chronic obesity is a risk factor for heart failure (HF), even without traditional heart disease risk factors, and it is an independent risk factor for death.^[3,4]^ Morbid obesity can cause structural changes in the heart, including increased left ventricular volume, left ventricular hypertrophy, left ventricular and left atrium dilatation, and in some cases, morbid obesity can cause diastolic and systolic dysfunction.^[5]^ The mechanisms behind these changes are still poorly understood. More recently, adipokines, such as leptin, resistin, and adiponectin, as well as gut hormones, such as glucagon-like peptide-1 and glucose-dependent insulinotropic polypeptide, have been postulated as mediators of the relationship between obesity and left ventricular systolic dysfunction (LVSD).^[6]^

Traditional approaches to weight loss, such as diet, lifestyle, and behavioral interventions, have been shown to be less effective in patients with morbid obesity and prone to rebound, especially when used alone.^[7]^ In terms of weight loss, diabetes, hypertension, or dyslipidemia improvement, bariatric surgery has become a safe, effective, and long-lasting treatment for morbid obesity.^[8]^

There are 3 main categories of bariatric surgery: restrictive surgery, malabsorptive surgery, and combination surgery. Restrictive surgery, such as gastric banding and sleeve gastrectomy, reduces the size of the stomach and leads to a feeling of fullness with less food, which ultimately leads to food intolerance, and weight loss. Malabsorptive surgeries, such as biliopancreatic diversion with or without duodenal switching and ileal interposition, bypass the intestinal segment, resulting in malabsorption of nutrients. Combined operations include both restriction and malabsorption, such as the Roux-en-Y gastric bypass.^[9]^

Although bariatric surgery has been reported to have a positive effect on improvements in cardiac geometry and function, it can also significantly improve left ventricular hypertrophy, diastolic dysfunction and cardiac load.^[10,11]^ However, the prognosis of patients with morbid obesity combined with cardiac systolic dysfunction after bariatric surgery is rarely reported. Here, we report a case of a patient with morbid obesity with LVSD whose cardiac function improved after bariatric surgery. This is the first case report in the Asia-Pacific region.

## 2 Case report

We performed a retrospective analysis of the clinical data of 1 morbidly obese patient with LVSD who underwent bariatric surgery in our hospital in November 2019. we collected data regarding patient demographics, HF etiology, and EF. The severity implication of the patients’ HF was also classified according to the New York Heart Association (NYHA) stage. Preoperative weight, postoperative weight, complications, body mass index (BMI), excess weight loss percentage, echocardiography, total weight lost percentage, and other data were recorded. The patient was successfully operated on with laparoscopic sleeve gastrectomy plus jejunal bypass (LSG+JJB) by an experienced surgical team. No intraoperative adverse events occurred, and no postoperative complications occurred. Moreover, the postoperative recovery was smooth. Because this report included a single case, statistical methods were limited to ranges and simple means. The protocol for this study was approved by the Ethics Committee of the hospital. Informed consent was not required for such reporting, but we obtained the patient's informed consent to publish the case.

The patient was a 36-year-old male with a height of 172 cm, weight of 135.8 kg, BMI of 45.9 kg/m^2^, blood pressure of 108/74 mm Hg, NYHA class IV, dilated cardiomyopathy (DCM) and left ventricular ejection faction (LVEF) of 31%. Moreover, the patient had hyperuricemia, hyperlipidemia, fatty liver disease, and severe sleep apnea. Preoperative data on the patient are presented in Table 1. The patient was admitted to the hospital due to progressive weight gain of more than 10 years. The patient used to be in good health. One year before admission, the patient was hospitalized in another hospital due to shortness of breath. After a relevant examination, the patient was diagnosed with DCM.

**Table 1:** Preoperative clinical condition of the patient.

Age	HF Type	NYHA Class	EF (%)	BMI (kg/m^2^)	Weight (kg)	EW (kg)
36 M	DCM	IV	31	45.9	135.8	68.8

BMI = body mass index, DCM = dilated cardiomyopathy, EF = ejection fraction, EW = excess weight, HF = heart failure, M = male, NYHA = New York Heart Association.

We followed up the patient regularly after surgery. One month after surgery, the patient data were as follows: weight of 114.1 kg, BMI of 38.67 kg/m^2^ and EF of 34%. Three months after surgery, the patient data were as follows: weight of 106.1 kg, BMI of 35.86 kg/m^2^ and EF of 40%. Six months after surgery, the patient data were as follows: weight of 102.2 kg, BMI of 34.54 kg/m^2^, EF of 55% and cardiac function recovery to NYHA class I. The ejection fraction returned to normal levels, and the patient's hyperuricemia, hyperlipidemia, and sleep apnea were significantly improved. Postoperative data of the patient are presented in Table 2. Preoperative echocardiography showed that the patient's whole heart was enlarged, and regular postoperative echocardiography showed that the enlargement of the right heart had recovered and that the enlargement of the left heart had improved. The echocardiography results are presented in Table 3.

**Table 2:** Postoperative clinical condition of the patient.

Follow-up (mo)	Weight (kg)	%TWL	%EWL	BMI (kg/m2)	BMI change	EF%	NYHA Class
1	114.1	15.98	31.54	38.67	7.23	31	III
3	106.1	21.87	43.17	35.86	10.04	40	II
6	102.2	24.74	48.84	34.54	11.36	55	I

BMI = body mass index, EF = ejection fraction, EWL = excess weight lost, NYHA = New York Heart Association, TWL = total weight lost.

**Table 3:** Echocardiographic results of the patient.

		Postoperative		
	Preoperative	1 mo	3 mo	6 mo
AO (mm)	29	31	29	31
LA (mm)	53	47	45	39
LVD (mm)	68	67	69	59
LVS (mm)	57	57	55	42
RA (mm)	46	40	37	38
RV (mm)	26	24	24	23
VS (mm)	11	9	9	11

AO = aortic diameter, LA = left atrial diameter, LVD = left ventricular end-diastolic diameter, LVS = left ventricular end-systolic diameter, RA = right atrium diameter, RV = right ventricle diameter, VS = ventricular septal thickness.

## 3 Discussion

The normal value of LVEF is 50% to 70%, and EF < 40% is diagnosed as HF with decreased ejection fraction.^[12]^ The patient's preoperative BMI and LVEF were 45.9 kg/m2 and 31%, respectively, and the patient was diagnosed with morbid obesity combined with HF. At the same time, the patient had DCM, hyperuricemia, hyperlipidemia, fatty liver disease, and severe sleep apnea. The relationship between obesity and the development of HF is complex and involves many different pathways that ultimately affect heart function. Morbid obesity not only affects cardiac hemodynamics due to changes in body mass and blood volume but also affects the structure and function of the heart due to disorders of the metabolic environment of obesity, including hyperleptinemia, hyperinsulinemia, and the activation of the renin angiotensin system. These changes may lead to increased left ventricular volume, left ventricular hypertrophy, left atrial enlargement and the development of diastolic, and systolic dysfunction.^[13,14]^ Moreover, morbidly obese patients often have diabetes, hypertension, obstructive sleep apnea, and metabolic syndrome, which may increase the risk of cardiac function obstacles.^[15]^

Bariatric surgery is associated with decreases in left and right ventricular mass, end-diastolic volume and diastolic dysfunction and increases in aortic distensibility,^[16]^ which is consistent with the results of echocardiography in this patient. Moreover, bariatric surgery can improve patients’ systolic function with HF. Ramani and Vest et al^[4,17]^ found that LVEF significantly improves after bariatric surgery in patients with LVSD. Due to the large heterogeneity of patients, small sample size, different surgical methods used, and the lack of a control group in many studies, there are significant differences in the reported improvements in cardiac contractile function after bariatric surgery. In the present report, the weight of the patient decreased by 33.6 kg 6 months after LSG+JJB, and the EF value increased from 31% to 55% at the same time, reaching the normal level, and cardiac function recovered to NYHA class I. These results suggest that bariatric surgery can safely and effectively improve LVSD in obese patients, but this conclusion needs to be confirmed with a larger sample size.

The mechanisms for the improvement of cardiac failure after bariatric surgery are unknown but likely involve a combination of multiple beneficial mechanisms.^[13]^ These mechanisms may include the effects of significant weight loss on cardiac workload and metabolism as well as beneficial alterations in the enterocardiac axis. First, significant weight loss reduces preload and afterload as well as systemic and local energy requirements. Myocardial oxygen consumption decreases significantly with weight loss and correlates with improved diastolic function.^[18]^ Other beneficial effects of bariatric surgery include changes in the gastrointestinal microbiome, increases in postprandial bile acids and increases in postoperative glucagon-like peptide-1 and receptor signaling.^[19]^ Bariatric surgery may be beneficial to alter the enterocardiac axis through 1 or more of these mechanisms, providing additional improvements in cardiac function in addition to weight loss.^[13]^ In this report, the patient lost only 3.9 kg in weight 3 to 6 months after surgery, but the EF of the patient increased from 40% to 55%. This result may suggest that bariatric surgery may improve systolic function in patients independent of weight loss. One study has shown that in a rodent model of sleeve gastrectomy and diet-induced cardiac dysfunction, sleeve gastrectomy significantly improves systolic function in 44% of rats in a weight loss-independent manner,^[20]^ supporting the case of postoperative cardiac function improvement independent of weight loss. However, the mechanism of independent improvement of cardiac function after bariatric surgery still needs further confirmation.

Bariatric surgery is a safe, effective, and durable treatment for patients with morbid obesity and systolic HF, and it can significantly improve ventricular systolic function. Thus, LSG+JJB may be used as a treatment for morbid obesity combined with HF. However, the mechanism of improved cardiac function after bariatric surgery still needs further study.

## Author contributions

**Conceptualization:** Huawu Yang.

**Data curation:** Dafang Zhan, Luo Fu, Jiahui Yu.

**Writing – original draft:** Pengsen Guo.

**Writing – review & editing:** Yanjun Liu.

## Abbreviations

Abbreviations: BMI = body mass index, DCM = dilated cardiomyopathy, HF = heart failure, LSG+JJB = laparoscopic sleeve gastrectomy plus jejunal bypass, LVEF = left ventricular ejection faction, LVSD = left ventricular systolic dysfunction, NYHA = New York Heart association.
